# Idiopathic Omental Infarction Presenting With Recurrent Abdominal Pain

**DOI:** 10.7759/cureus.33796

**Published:** 2023-01-15

**Authors:** Maram Nached, Yasmin Nached, Arfan Al Awa

**Affiliations:** 1 General Surgery, Dubai Health Authority, Dubai, ARE; 2 General Surgery, Dubai Medical College for Girls, Dubai, ARE

**Keywords:** omentum, computed tomography abdomen, acute abdomen, meckels diverticulum, omental infarction

## Abstract

Omental infarction is a rare cause of acute abdominal pain, and its rarity is mainly due to its relatively rich blood supply by multiple collateral vessels. It usually presents with right lower quadrant pain, as left-sided torsion is infrequent and is usually diagnosed intraoperatively.

Since omental infarction is frequently diagnosed by CT scan, conservative management should be considered in most patients to avoid subjecting the patients to unnecessary surgical intervention.

We present a rare case of idiopathic omental infarction in which the patient was initially radiologically diagnosed with Meckel's diverticulitis but was later found to have omental infarction on diagnostic laparoscopy.

## Introduction

Omental infarction due to torsion is a rare condition, and idiopathic infarction is furthermore rare; only 400 cases of omental infarction have been reported in the literature [1]. Typically, it presents with pain in the lower or upper quadrant, which usually mimics appendicitis or cholecystitis, and the usual population involves children and males with morbid obesity [2]. Management would include conservative treatment if the tumor is correctly diagnosed by imaging and the patient's condition is stable; if not, then laparoscopic intervention would be appropriate. Therefore, early recognition can reduce morbidity and unnecessary invasive workup and treatment [3,4]. However, in our case, the patient was initially diagnosed with Meckel’s diverticulum and persistent abdominal pain, so she was taken for a diagnostic laparoscopy. Thus, it is important to report such cases in order to add to the limited body of knowledge in the literature.

## Case presentation

A 17-year-old previously healthy male presented to the emergency room with a two-day history of abdominal pain. The pain started in the right lower quadrant, then became more generalized, with no history of fever, nausea, or vomiting, no changes in bowel habits, or a loss of appetite. On further assessment, the patient's vital signs were normal, his abdomen was lax with mild tenderness in the periumbilical area, and his labs were as shown in Table 1.

**Table 1:** Labs

Lab test	Result		Reference range
	First visit	Second visit (2 weeks later)	
WBC	10.2 × 10^3^/µL	11.2 × 10^3^/µL	3.6–11 × 10^3^/µL
Neutrophil absolute count	6.7 × 10^3^/µL	9.4 × 10^3^/µL	2.0-7.0 *10^3^/µL
Haemoglobin	14.0 g/dL	14.4 g/dL	13–17 g/dL
Creatinine	0.5 mg/dL	0.6 mg/dL	0.7–1.2 mg/dL
CRP	4.4 mg/L	1.3 mg/L	<0.5 mg/L
Lactic acid	3.3 mol/L	2.7 mol/L	0.5–2.2 mol/L
Lipase	14 U/L	16 U/L	13–60 U/L

**Figure 1:**
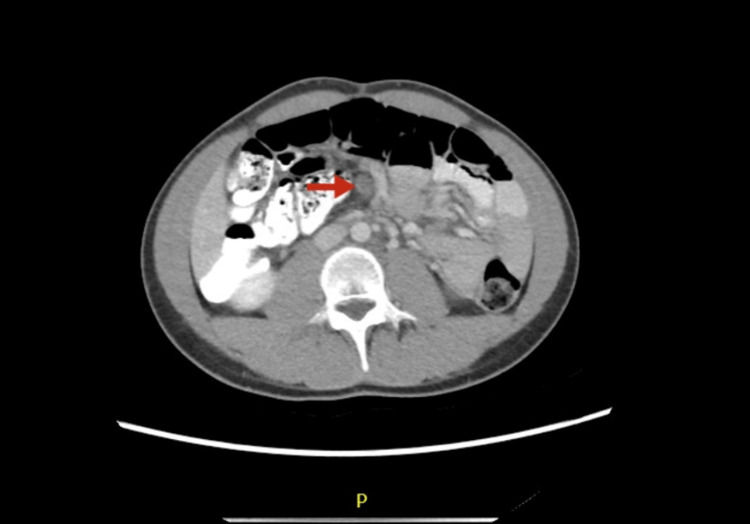
Tubular lesion with enhancing walls in centre of abdomen with surrounding fat stranding.

To complete his evaluation, a computed tomography scan (shown in Figure 1) was done in view of the suspicion of acute appendicitis, which showed a tubular lesion with mildly enhanced walls in the central abdomen and peri-lesional fat stranding with a differential of Meckel’s diverticulitis or panniculitis.

Based on this, the patient was discharged from the emergency department on oral antibiotics and advised for clinic follow-up. However, seven days later, the patient presented again to the emergency room with the same complaint, was managed conservatively, and was discharged by an emergency physician.

On the follow-up visit to the clinic, the patient was doing well and was advised to follow up after three months. However, one-week later patient presented again to the emergency room with a similar presentation, severe ON/OFF periumbilical and right lower quadrant pain, abdomen lax with tenderness in the right lower quadrant with a rebound. In this presentation, the results of the labs are shown in Table 1.

**Figure 2:**
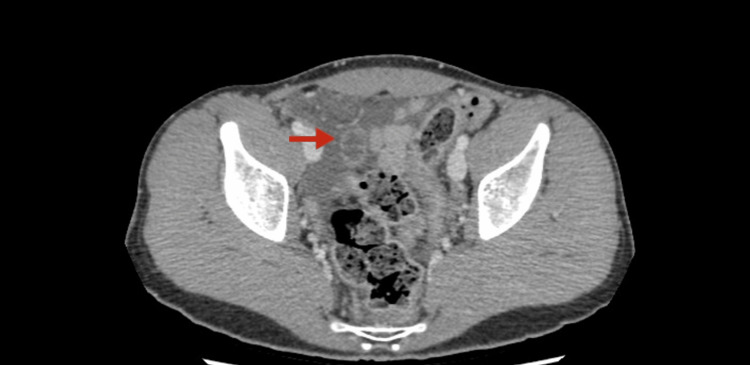
Peripherally enhancing rounded cystic structure (arrow) with mesenteric lymphadenopathy.

A repeat CT scan (shown in Figure 2) showed pelvic-free fluid associated with a lower abdominal peripherally enhanced rounded cystic structure closely related to the small bowel loop and mesenteric lymphadenopathy, suggestive of Meckel’s diverticulitis.

The patient and his family were offered admission and a diagnostic laparoscopy. The intraoperative findings included a moderate amount of turbid fluid in the pelvis; the appendix was normal; prominent mesenteric lymph nodes; an area of omental infarction (4 cm × 4 cm without torsion), which was resected; and a small bowel was run without any gross pathology detected. Therefore, a diagnosis of omental infraction was made, and an omentectomy was done for the ischemic part, and the fluid was sent for culture. The patient postoperatively was doing well, started on diet gradually, and was discharged on day 2 postoperatively with a follow-up appointment in the clinic. Later, the fluid culture was negative, and histopathology proved omental hemorrhagic infarction. The patient was reassured and discharged from the general surgery clinic.

## Discussion

The great omentum is a double fold of peritoneum that extends from the greater curvature of the stomach to the transverse colon. It is regarded as the policeman of the abdomen as it is rich in lymphatic tissue, and therefore its role is to defend against intra-abdominal infection [5].

Omental infarction is a rare cause of abdominal pain; almost 400 cases have been reported in the literature [1]. The usual etiology of infarction is due to torsion, which would result in cessation of blood supply to that area and necrosis.

Torsion of the omentum can be either primary, where no other intra-abdominal pathology could be identified, or secondary infarction, in which case a pathology could be identified that could act as a lead point for torsions like adhesions, tumors, or hernias. However, in rare cases, omental infarction could be idiopathic without torsion, in which case no clear mechanism can be identified. Some case reports have regarded abdominal trauma, mesenteric artery occlusion, obesity, or congestive heart failure as possible causes of idiopathic omental infarction [6]. However, in our case, no identifiable pathology or precipitating factor was found, and it was therefore regarded as an idiopathic omental infarction.

Diagnosis of omental infarction is quite challenging as it mimics features of acute abdomen, CT abdomen therefore would be the imaging of choice to exclude other causes of acute abdomen, including acute appendicitis, diverticulitis, epiploic appendagitis, and perforation [7].

The expected CT features in omental infarction would include a focal area of fat stranding and swirling of omental vessels in omental torsion with a hyperdense peripheral halo [8]. However, in our case, no signs of torsion were seen; instead, a peripherally enhanced rounded cystic structure closely related to the small bowel loop was reported as suggestive of Meckel’s diverticulum.

There has been controversy in the management of omental infarction with no specific guidelines in the literature, and thus it should be decided individually based on the clinical findings and imaging [9].

Conservative management includes oral analgesics, anti-inflammatory medications, and antibiotics. The advantage of early surgical intervention is the reduced incidence of necrosis, abscess formation, and adhesion formation. Laparoscopic surgery can also decrease the patient's time in the hospital [10]. In our case, the patient was in severe, uncontrolled pain with inconclusive CT features of Meckel's diverticulitis and was therefore taken for a diagnostic laparoscopy.

## Conclusions

After ruling out other differentials, omental infarction should be considered as a differential in patients presenting with acute abdomen. Specific CT scan features can help in supporting the diagnosis, and management would be case-based. In our case, CT features were not supportive, and our previously healthy 17-year-old patient was successfully managed by a laparoscopic approach due to persistent pain.
